# Crystal structure of tyrosine-tRNA synthetase from *Helicobacter pylori* G27

**DOI:** 10.1107/S2053230X26010253

**Published:** 2026-10-23

**Authors:** David Igbalajobi, Jason E. Taylor, Raymond Gao, Jesuferanmi Ayanlade, Rachael Zigweid, Sandhya Subramanian, Hannah Udell, Thomas E. Edwards, Jan Abendroth, Donald Lorimer, Peter J. Myler, Bart Staker, Edmund Moses Ndip, Oluwatoyin A. Asojo

**Affiliations:** a Dartmouth College Hanover New Hampshire USA; b University of California, Berkeley, Berkeley, California, USA; c Center for Global Infectious Disease Research Seattle Children’s Research Institute 307 Westlake Avenue North, Suite 500 Seattle WA98109 USA; d Seattle Structural Genomics Center for Infectious Diseases, Seattle, Washington, USA; e UCB BioSciences, Bainbridge Island, Washington, USA; f Chemistry and Biochemistry Hampton University Hampton VA23666 USA; g Dartmouth Cancer Center, Lebanon, New Hampshire, USA; h Biochemistry and Cell Biology, Dartmouth Geisel School of Medicine, New Hampshire, USA; Bristol-Myers Squibb, USA

**Keywords:** SSGCID, gastric cancer, tyrosine-tRNA synthetase, *Helicobacter pylori*, drug repurposing

## Abstract

*Helicobacter pylori*, a major cause of gastric ulcers and cancers, infects nearly half of the world’s population. The crystal structure of a potential *H. pylori* structure-guided drug-discovery target, tyrosine-tRNA synthetase (*Hp*TyrRS), has been determined by the Seattle Structural Genomics Center for Infectious Disease (SSGCID). *Hp*TyrRS is an enzyme that catalyzes the ATP-dependent attachment of tyrosine to tRNA. *Hp*TyrRS was produced and crystallized, and its structure was determined at 2.2 Å resolution. *Hp*TyrRS has a prototypical bacterial TyrRS topology and forms dimers in solution. *Hp*TyrRS shares a conserved binding cavity for a previously identified *Staphylococcus aureus* TyrRS inhibitor (SB-219383). The reported structure and analysis establish *Hp*TyrRS as a potential target for structure-guided drug discovery and repurposing.

## Abstract 2

The production, crystallization and 2.2 Å resolution structure of *H. pylori* tyrosine-tRNA synthetase are reported.

## 1. Introduction

*Helicobacter pylori* is a helical, Gram-negative, microaerophilic bacterium that colonizes the stomach of ∼50% of the world’s human population (Costa *et al.*, 2024 ▸). *H. pylori* causes chronic gastritis, peptic ulcer disease (PUD), gastric mucosa-associated lymphoid tissue lymphoma (MALT) and gastric adenocarcinoma (Sabbagh *et al.*, 2019 ▸). The helical shape of *H. pylori* facilitates movement in the human body, while the coccoid form allows colonization of the gastric epithelial cells (Al-Fakhrany & Elekhnawy, 2023 ▸). *H. pylori* colonization induces pathological changes in the gastric microenvironment (Wang *et al.*, 2015 ▸). Increasing antibiotic resistance of infectious bacteria has been a global concern for decades, prompting the adoption of combination therapy involving two or more types of antibiotics, such as amoxicillin, clarithromycin, metronidazole, tetracycline, levofloxacin and rifabutin, as well as bismuth, to enhance healing and protect the gastric mucosa (Wang *et al.*, 2015 ▸). The increasing drug resistance of *H. pylori* reveals an unmet need for new therapeutics. The Seattle Structural Genomics Center for Infectious Disease (SSGCID) has identified *H. pylori* aminoacyl-tRNA synthetases, including tyrosine-tRNA synthetase (*Hp*TyrRS), as targets for structure-guided drug discovery and repurposing. TyrRS catalyzes the bonding of tyrosine to tRNA (Xin *et al.*, 2000 ▸). Aminoacyl-tRNA synthetases (aaRSs) are enzymes that catalyze the esterification of amino acids to the corresponding tRNA, where the product of the process plays a crucial role in protein synthesis (Rubio Gomez & Ibba, 2020 ▸). The aaRS family, specifically TyrRS, have been identified as promising targets for new antibacterials (Zhu *et al.*, 2015 ▸; Sun *et al.*, 2017 ▸; Monobe *et al.*, 2024 ▸). Here, we present the purification, crystallization and structure of *Hp*TyrRS as a first step towards structure-guided inhibitor design.

## 2. Materials and methods

### 2.1. Macromolecule production

*Hp*TyrRS was cloned, expressed and purified using previously described methods (Stacy *et al.*, 2011 ▸; Serbzhinskiy *et al.*, 2015 ▸; Rodríguez-Hernández *et al.*, 2023 ▸). The full-length gene for tyrosine-tRNA synthetase (EC 6.1.1.1) from *H. pylori* G27 (UniProt B5Z7D7) encoding amino acids 1–402 was PCR-amplified from genomic DNA using the primers in Table 1 ▸. The gene was cloned into the expression vector BG1861 to generate N-terminally His-tagged plasmid DNA. The plasmid was transformed into chemically competent *Escherichia coli* BL21(DE3) Rosetta cells. These cells are an institutional variation of the classic Rosetta expression strain that layer a protective phage-resistance mutation onto the rare-codon translation power of the Rosetta strain.

After small-scale expression testing, a 2 l culture was grown in a LEX Bioreactor (Epiphyte Three) using auto-induction media (Studier, 2005 ▸) and standardized SSGCID methods (Serbzhinskiy *et al.*, 2015 ▸). *Hp*TyrRS was purified using the previously described two-step protocol of an immobilized metal (Ni^2+^)-affinity chromatography (IMAC) step followed by size-exclusion chromatography (SEC) on an ÄKTApurifier 10 (GE Healthcare) using automated IMAC and SEC programs (Serbzhinskiy *et al.*, 2015 ▸). Briefly, thawed bacterial pellets (25 g) were lysed by sonication in 200 ml lysis buffer [20 m*M* HEPES pH 7.0, 300 m*M* NaCl, 5%(*v*/*v*) glycerol, 0.5%(*w*/*v*) CHAPS, 30 m*M* imidazole, 10 m*M* MgCl_2_ and five tablets of protease inhibitor cocktail (cOmplete Mini, EDTA-free Roche, Basel, Switzerland)]. After sonication, the crude lysate was incubated with 20 µl (25 units per µl) benzonase while mixing for 45 min at room temperature. The lysate was centrifuged at 5000*g* for 1 h to remove insoluble debris. The clarified supernatant was passed over an Ni–NTA HisTrap FF 5 ml column (GE Healthcare), which had been pre-equilibrated with wash buffer [20 m*M* HEPES pH 7.0, 300 m*M* NaCl, 5%(*v*/*v*) glycerol, 30 m*M* imidazole]. The column was washed with 20 column volumes (CV) of wash buffer and eluted with elution buffer [20 m*M* HEPES pH 7.0, 300 m*M* NaCl, 5%(*v*/*v*) glycerol, 500 m*M* imidazole] over a 7 CV linear gradient. Peak fractions were pooled and concentrated to a final volume of 5 ml for SEC. The 5 ml protein sample was loaded onto a Superdex 75 26/60 column (GE Biosciences) attached to an ÄKTAprime plus FPLC system (GE Biosciences) that had been pre-equilibrated with SEC buffer (20 m*M* HEPES pH 7.0, 300 m*M* NaCl, 5% glycerol, 1 m*M* TCEP). *Hp*TyrRS eluted as a single, symmetrical, monodisperse peak. The peak fractions were collected and assessed for purity by SDS–PAGE on a 4–20% protein gel (Invitrogen) and visualized by staining with InstantBlue colloidal stain (Expedeon, San Diego, California. USA). Recombinant *Hp*TyrRS was ∼95% pure. The peak fraction was pooled and concentrated to ∼20 mg ml^−1^ using an Amicon purification system (Millipore). Aliquots were flash-frozen in liquid nitrogen and stored at −80°C until use.

### 2.2. Crystallization

*Hp*TyrRS was screened for crystallization conditions using the Molecular Dimensions Morpheus commercial screen. Crystals were grown in Molecular Dimensions Morpheus condition D11 (Table 2 ▸) and did not require additional cryoprotectant prior to data collection.

### 2.3. Data collection and processing

Data were collected as reported in Table 3 ▸. Data were integrated with *XDS* and reduced with *XSCALE* (Kabsch, 2010 ▸). Raw X-ray diffraction images are stored at the Integrated Resource for Reproducibility in Macromolecular Crystallography at https://www.proteindiffraction.org.

### 2.4. Structure solution and refinement

The structure of *Hp*TyrRS was determined by molecular replacement with *SIMBAD* (Simpkin *et al.*, 2018 ▸) from the *CCP*4 suite of programs (Collaborative Computational Project, 1994 ▸; Krissinel & Henrick, 2004 ▸; Winn *et al.*, 2011 ▸;Agirre *et al.*, 2023 ▸) using PDB entry 1h3f (Yaremchuk *et al.*, 2002 ▸) as the search model. The structure was refined using iterative cycles of *phenix.refine* (Afonine *et al.*, 2012 ▸) followed by manual rebuilding of the structure using *Coot* (Emsley & Cowtan, 2004 ▸; Emsley *et al.*, 2010 ▸).

**Figure 1:**
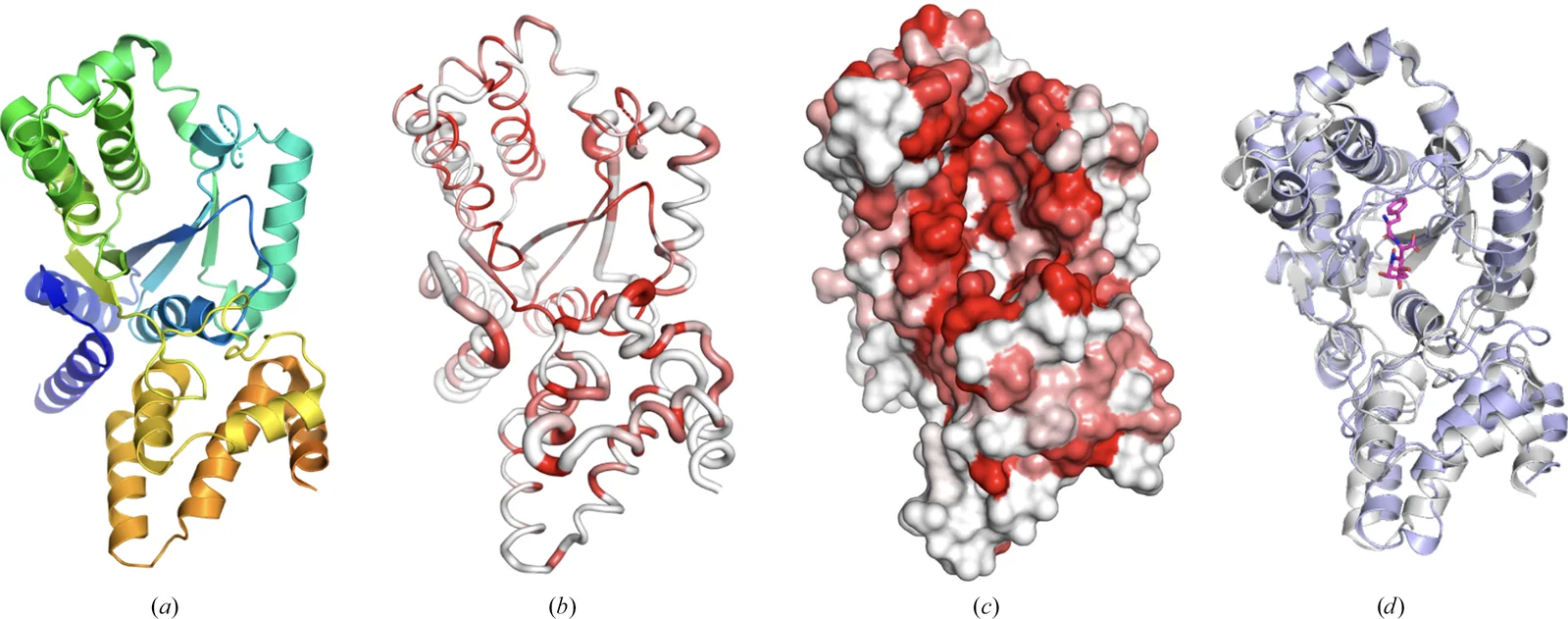
*Hp*TyrRS structure. (*a*) *Hp*TyrRS has a prototypical TyrRS topology (colored from blue at the N-terminus to red at the C-terminus). (*b*) *ENDscript* ribbon diagram. Ribbon thickness reflects secondary-structure similarity: thinner ribbons indicate more conserved regions and thicker ribbons suggest lower conservation, as determined by r.m.s.d. alignment of structures. The ribbon is colored by sequence identity (red = identical residues). (*c*) Solvent-accessible surface area of *Hp*TyrRS colored by *ENDscript* sequence conservation, with identical residues colored red, reveals a large conserved central cavity. (*d*) SB-219383 in magenta sticks sits in the open central cavity in both *Sa*TyrRS (blue) and *Hp*TyrRS (gray). *Hp*TyrRS (PDB entry 6byq) was aligned with *Sa*TyrRS (PDB entry 1jii) within *PyMOL* to generate (*d*). (*a*)–(*d*) are in the same orientation. All figures were generated with *PyMOL*.

One molecule is present in the asymmetric unit, consistent with a Matthews coefficient of 2.74 Å^3^ Da^−1^, corresponding to a 55% solvent content in the protein crystal. The final model included two N-terminal histidine residues from the His-tag and 311 main-chain residues (Fig. 1 ▸, Table 4 ▸). Six vector-derived residues and 96 main-chain residues were not modeled because they lacked unambiguous electron density at a 0.9 r.m.s.d. cutoff in the 2*F*_o_ − *F*_c_ electron density after iterative cycles of model building and refinement. The structure quality was checked using *MolProbity* (Williams *et al.*, 2018 ▸). Refinement statistics are shown in Table 4 ▸. Coordinates and structure factors have been deposited with the Worldwide PDB (wwPDB) as entry pdb_00006byq.

### 2.5. Inhibitor docking

Inhibitor docking was performed using *AutoDock Vina* v1.2.7 to determine whether *Hp*TyrRS is suitable for drug repurposing (Eberhardt *et al.*, 2021 ▸; Cosconati *et al.*, 2010 ▸; Trott & Olson, 2010 ▸).* UCSF Chimera DockPrep* was used to prepare the ligand, add hydrogens and charges, remove solvent molecules and repair incomplete side chains in *Hp*TyrRS (Lang *et al.*, 2009 ▸; Moustakas *et al.*, 2006 ▸). The docking search space was defined based on residues within 5 Å of SB-219383 in the predicted active site, after structural alignment with *Staphylococcus aureus* TyrRS (*Sa*TyrRS; PDB entry 1jii; Qiu *et al.*, 2001 ▸) within *PyMOL*. The grid box was centered at *X* = −17.1375 Å, *Y* = −20.2976 Å, *Z* = 14.9006 Å, with box dimensions of 17.6119 × 23.3816 × 26.8822 Å and a grid spacing of 0.375 Å. Docking simulations were conducted with an exhaustiveness of 12, using 8 CPU cores and a verbosity level of 1. As a control, the docking search was repeated with *Sa*TyrRS.

## 3. Results and discussion

Preparative SEC revealed that *Hp*TyrRS assembles as a ∼77 kDa dimer, consistent with *PISA* analysis (https://www.ebi.ac.uk/pdbe/pisa/). This dimer suggests a monomer of ∼38 kDa, which is smaller than the expressed protein of 45 kDa. Approximately 24% of the sequence was missing and could not be modeled, notably 83 residues at the carboxyl terminus from Gln319 through Asn402 (Table 1 ▸). Additionally, six vector-derived residues ^−8^MAHHHH^−3^ and seven residues ^86^DPTGKNE^92^ could not be modeled. The expected molecular weight of the modeled protein is ∼37 kDa, consistent with SEC data. A possible hypothesis is C-terminal proteolysis. Mass-spectroscopic analysis of recombinant *Hp*TyrRS and *Hp*TyrRS crystals is planned to confirm their amino-acid sequence and predicted molecular mass.

*Hp*TyrRS is a prototypical bacterial TyrRS with the canonical bacterial tyrosine-tRNA (TyrRS) architecture consisting of a compact, globular α/β fold comprising 19 α-helices and six β-strands, organized into three βαβ units, one β-bulge and a single β-sheet (Fig. 1 ▸). *ENDscript* analysis (Gouet *et al.*, 1999 ▸, 2003 ▸) revealed that *Hp*TyrRS shares extensive secondary-structural similarity with other bacterial TyrRS, and the closest structural homologs of *Hp*TyrRS are TyrRS from *Acinetobacter baumannii* (PDB entry 6bqy), *Thermus thermophilus* (PDB entry 1h3e) and *E. coli* (PDB entry 1vbm) (Yaremchuk *et al.*, 2002 ▸; Kobayashi *et al.*, 2005 ▸; Figs. 1 ▸*b*, 1 ▸*c* and Supplementary Fig. S1). Similarly, *PDBeFold* analysis (https://www.ebi.ac.uk/msd-srv/ssm; Krissinel & Henrick, 2004 ▸) with a default threshold cutoff of 70% identified the closest structural neighbor of *Hp*TyrRS as the *A. baumannii* TyrRS structure (PDB entries 6bqy and 6bqz), with ∼57% sequence identity and ∼1.6 Å r.m.s.d for alignment of all main-chain atoms. Likewise, a *DALI* search for similar structures against the PDB was consistent with *PDBeFold* and established *Hp*TyrRS as a prototypical bacterial TyrRS, supporting the feasibility of inhibitor repurposing from other bacterial TyrRS.

**Figure 2:**
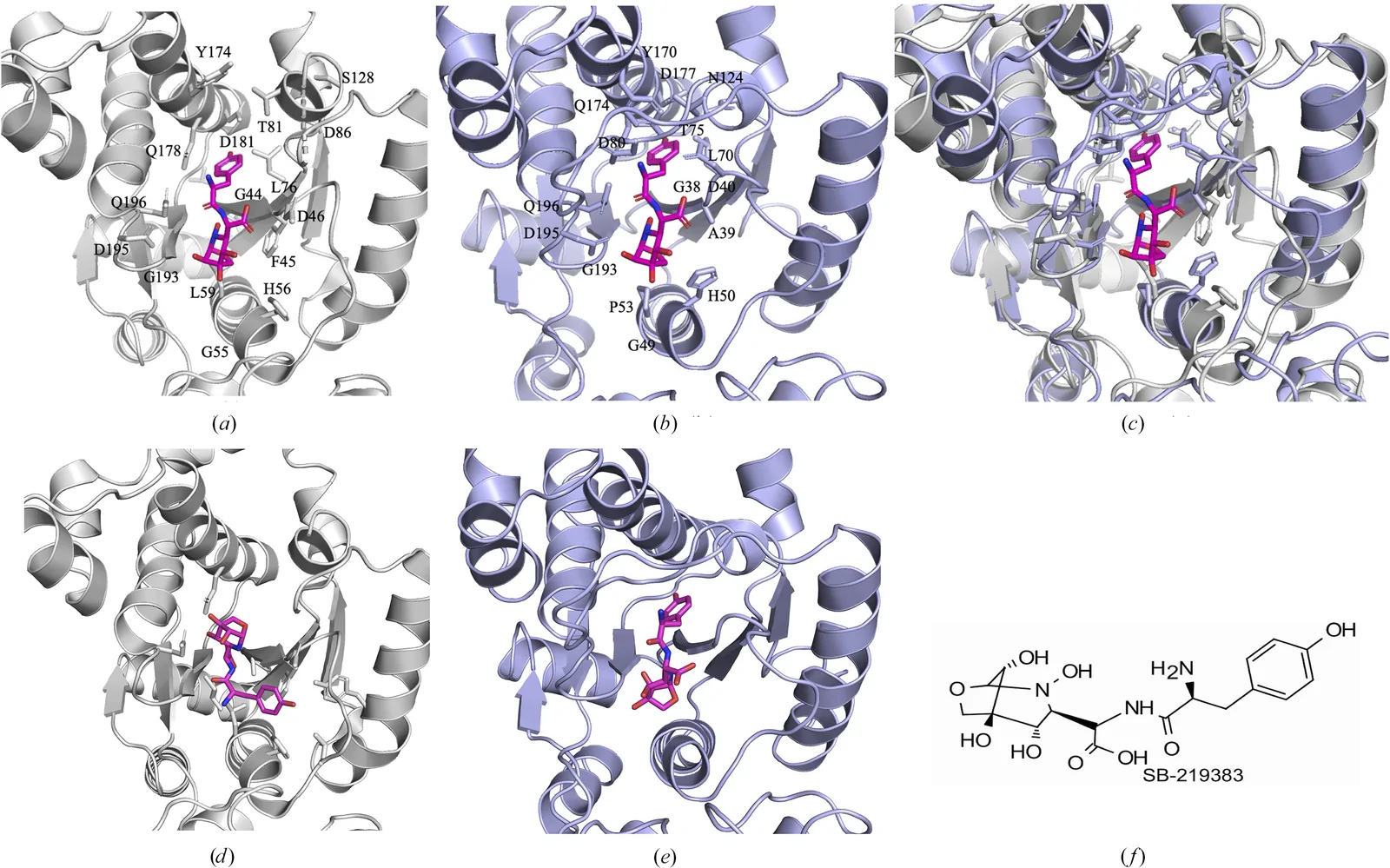
Superposed and modelled SB-219383 inhibitor binding. (*a*) HpTyrRS has conserved amino-acid residues in the superposed SB-219383 binding cavity. (*b*) SB-219383 (magenta) binds to *Sa*TyrRS (blue) residues shown as blue sticks, as identified from PDB entry 1jii. (*c*) Superposed *Hp*TyrRS (gray) and *Sa*TyrRS (blue) reveal conserved amino acids. (*d*) Top docking pose of SB-219383 with HpTyrRS; docking score −8.03. (*e*) Top docking pose of SB-219383 with *Sa*TyrRS; docking score −8.28. (*f*) *CHEMDRAW* representation of SB-219383. *Hp*TyrRS (PDB entry 6byq) was superposed with *Sa*TyrRS (PDB entry 1jii) within *PyMOL* to generate (*a*)–(*c*). (*a*)–(*e*) are in the same orientation as Fig. 1 ▸ and are magnified to visualize SB-219383. All figures were generated with *PyMOL* unless otherwise stated.

To assess the feasibility of future drug repurposing of *Hp*TyrRS, (2*S*)-2-[[(2*S*)-2-amino-3-(4-hydroxyphenyl)propanoyl]amino]-2-[(1*S*,3*S*,4*S*,5*R*,8*R*)-2,4,5,8-tetrahydroxy-7-oxa-2-azabicyclo[3.2.1]octan-3-yl]acetic acid; SB-219383; Fig. 2 ▸*d*), a competitive, selective and potent inhibitor of bacterial TyrRS, was docked to *Hp*TyrRS. As a control, SB-219383 was docked back into *Sa*TyrRS. SB-219383 is a potent (IC_50_ of ∼0.5 n*M*) *Sa*TyrRS inhibitor produced by *Micromonospora*, and a weaker inhibitor of mammalian TyrRS, specifically with an IC_50_ of ∼2 µ*M* for the rat liver orthologue (Stefanska *et al.*, 2000 ▸; Greenwood & Gentry, 2002 ▸). This selectivity profile strengthens the rationale for testing SB-219383 against *Hp*TyrRS.

**Figure 3:**
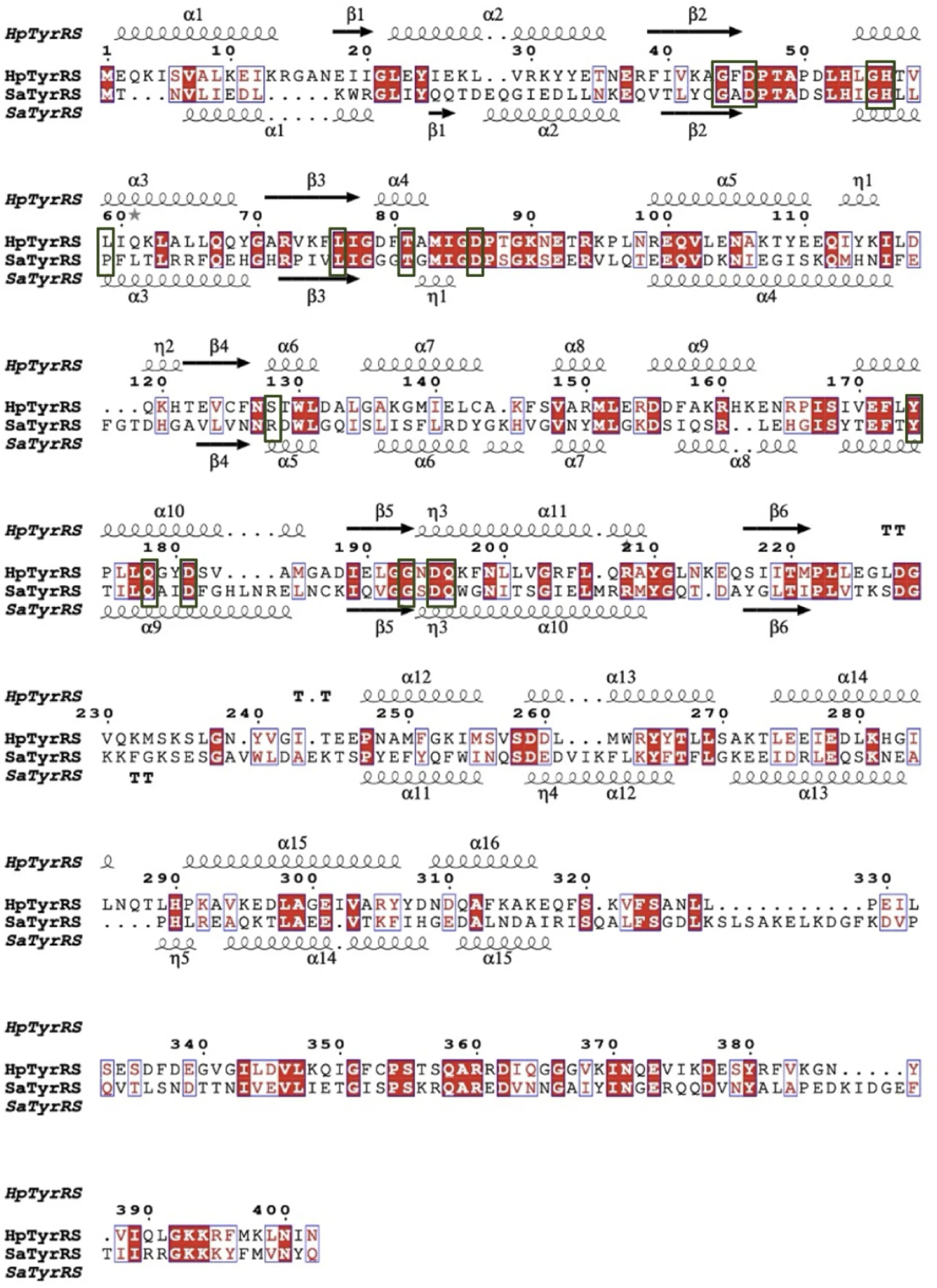
Structure-based primary-sequence alignment of *Hp*TyrRS (PDB entry 6byq) and *Sa*TyrRS (PDB entry 1jii). The secondary-structure elements are as follows: α-helices are shown as large coils, 3_10_-helices as small coils labeled η and β-strands as arrows labeled β, with β-turns labeled TT. Identical residues are shown in white font on a red background, while conserved residues are shown in red font and conserved regions are shown in blue boxes. The residues interacting with SB-219383 are indicated with green boxes. This figure was generated using *ESPript* 3.0 with alignment in *Clustal Omega*.

The structure of *Sa*TyrRS with bound SB-219383 has been reported as PDB entry 1jii (Qiu *et al.*, 2001 ▸). *Sa*TyrRS shares ∼26% sequence identity with *Hp*TyrRS and modest structural alignment (r.m.s.d. 2.9 Å, alignment length 295 residues; Fig. 1 ▸*d*). SB-219383 sits in a central binding cavity in *Sa*TyrRS (Figs. 1 ▸*d*, 2 ▸*b* and 2 ▸*c*). The central binding cavity of *Sa*TyrRS shares similar amino acids with *Hp*TyrRS (Figs. 2 ▸*a*–2 ▸*c*). Many residues within 5 Å of SB-219383 are conserved (Fig. 3 ▸ and Table 5 ▸). Additionally, some of these residues are conserved in bacterial TyrRS (Supplementary Fig. S1). The top docking pose of SB-219383 into *Hp*TyrRS (Fig. 2 ▸*d*) does not resemble the superposed orientation (Fig. 2 ▸*a*). The control docking of SB-219383 into *Sa*TyrRS (Fig. 2 ▸*e*) more closely resembles the crystal structure (Fig. 2 ▸*b*) than the top pose obtained from docking into *Hp*TyrRS (Fig. 2 ▸*d*), which may reflect the cavity’s large open size and suggest the need for more experimental data to define inhibitor binding by *Hp*TyrRS.

## 4. Conclusion

*Hp*TyrRS is a prototypical bacterial TyrRS with a large central pocket. Computational docking suggests that the pocket may be capable of binding validated *Sa*TyrRS inhibitors such as SB-219383 and may facilitate structure-guided *H. pylori* drug discovery and repurposing. Future studies will determine the activity of *HpTyrRS* and assess inhibition by known bacterial TyrRS inhibitors including SB-219383.

## Supplementary Material

PDB reference: tyrosine-tRNA synthetase from *Helicobacter pylori* G27, 6byq

Supplementary Fig S1. DOI: 10.1107/S2053230X26010253/rf5052sup1.pdf

Raw X-ray diffraction images: https://proteindiffraction.org/project/6byq/

## Figures and Tables

**Table 1:** Macromolecule-production information

Source organism	*Helicobacter pylori* (strain G27)
DNA source	Nina Salama, FHCRC
Forward primer	5′-CTCACCACCACCACCACCATATGGAACAAAAAATCAGTGTGGCC-3′
Reverse primer	5′-ATCCTATCTTACTCACTTAGTTGATATTTAATTTCATAAATCTTTTCT-3′
Expression vector	BG1861
Expression host	*Escherichia coli* BL21(DE3) R3 Rosetta
Complete amino-acid sequence of the construct produced†	**MAHHHH**HHMEQKISVALKEIKRGANEIIGLEYIEKLVRKYYETNERFIVKAGFDPTAPDLHLGHTVLIQKLALLQQYGARVKFLIGDFTAMIG**DPTGKNE**TRKPLNREQVLENAKTYEEQIYKILDQKHTEVCFNSTWLDALGAKGMIELCAKFSVARMLERDDFAKRHKENRPISIVEFLYPLLQGYDSVAMGADIELGGNDQKFNLLVGRFLQRAYGLNKEQSIITMPLLEGLDGVQKMSKSLGNYVGITEEPNAMFGKIMSVSDDLMWRYYTLLSAKTLEEIEDLKHGILNQTLHPKAVKEDLAGEIVARYYDNDQAFKAKE**QFSKVFSANLLPEILSESDFDEGVGILDVLKQIGFCPSTSQARRDIQGGGVKINQEVIKDESYRFVKGNYVIQLGKKRFMKLNIN**

† Additional residues from the His-tag are underlined. Disordered residues are shown in bold. Six vector-derived residues ^−8^MAHHHH^−3^ are unmodelled. Seven residues ^86^DPTGKNE^92^ and the C-terminal residues from ^319^Q through ^402^N are also disordered and unmodelled.

**Table 2:** Crystallization

Method	Vapor diffusion, sitting drop
Plate type	Tray 101-d6, 96-well plates
Temperature (K)	290
Protein concentration (mg ml^−1^)	23.6
Buffer composition of protein solution	20 m*M* HEPES pH 7.0, 300 m*M* NaCl, 5% glycerol, 1 m*M* TCEP
Composition of reservoir solution	10%(*w*/*v*) PEG 4000, 20%(*v*/*v*) glycerol, 0.02 *M* 1,6-hexanediol, 1-butanol, 0.02 *M* (*RS*)-1,2-propanediol, 0.02 *M* 2-propanol, 0.02 *M* 1,4-butanediol, 0.02 *M* 1,3-propanediol, 100 m*M* Bicine/Tris base pH 8.5
Volume and ratio of drop	0.4 µl, 1:1
Volume of reservoir (µl)	80

**Table 3:** Data collection and processing Values in parentheses are for the outer shell. Raw X-ray diffraction images have been deposited at https://proteindiffraction.org/project/6byq/. Data processing shows that ice rings could occur at 3.897, 3.669, 3.441, 2.671, 2.249, 2.072, 1.948, 1.918, 1.883 and 1.721 Å.

Diffraction source	APS beamline 21-ID-G
Temperature (K)	100
Detector	RayoniX MX-300 CCD
Space group	*C*2
Mosaicity (°)	0.146
Detector distance (mm)	325
Oscillation width (°)	1.00
Total rotation (°)	200
No. of frames	200
Wavelength (Å)	0.97872
*a*, *b*, *c* (Å)	121.40, 43.36, 113.70
α, β, γ (°)	90, 121.21, 90
Resolution range (Å)	40.01–2.24 (2.26–2.24)
Completeness (%)	97.1 (84.8)
No. of reflections	106143 (6118)
No. of unique reflections	25454 (1646)
Multiplicity	4.17 (3.74)
〈*I*/σ(*I*)〉	18.0 (2.6)
CC_1/2_	0.99 (0.79)
*R*_r.i.m._	0.056 (0.58)
Overall *B* factor from Wilson plot (Å^2^)	40

**Table 4:** Structure refinement Values in parentheses are for the outer shell. Validation and Ramachandran statistics were obtained with *MolProbity* within the wwPDB X-ray validation reports. Quality statistics are calculated using standard compilations of covalent geometry parameters (Engh & Huber, 2006 ▸; Parkinson *et al.*, 1996 ▸), tools in *MolProbity* (Chen *et al.*, 2010 ▸) and the wwPDB Chemical Component Dictionary. The majority of unmodeled residues are at the carboxyl-terminus.

Resolution range (Å)	40.01–2.20 (2.26–2.20)
Completeness (%)	97.3
σ Cutoff	*F* > 1.36σ(*F*)
No. of reflections, working set	25445 (1434)
No. of reflections, test set	1964 (139)
Final *R*_cryst_	0.178 (0.228)
Final *R*_free_	0.209 (0.257)
No. of non-H atoms	
Protein	2366
Ligand (IPA)	4
Solvent	123
Total	2493
R.m.s. deviations	
Bond lengths (Å)	0.008
Angles (°)	1.2
Average *B* factors (Å^2^)	
Protein	60
Ligand (IPA)	50
Water	60
Ramachandran plot	
Most favored (%)	98
Allowed (%)	2
Unmodelled/incomplete residues (%)	24
Modeled/analyzed (%)	76

**Table 5:** Residues within 5 Å of SB-219383

*Sa*TyrRS	*Hp*TyrRS
Gly38	Gly44
Asp40	Asp46
Gly49	Gly55
His50	His56
Leu70	Leu76
Thr75	Thr81
Asp80	Asp86
Asn124	Ser128
Tyr170	Tyr174
Gln174	Gln178
Asp177	Asp181
Gly193	Gly193
Asp195	Asp195
Gln196	Gln196
Tyr36	Lys42
Cys37	Ala 43
Ala 39	Phe45
Pro53	Leu59
Gln190	Glu190

## References

[bb1] Afonine, P. V., Grosse-Kunstleve, R. W., Echols, N., Headd, J. J., Moriarty, N. W., Mustyakimov, M., Terwilliger, T. C., Urzhumtsev, A., Zwart, P. H. & Adams, P. D. (2012). *Acta Cryst.* D**68**, 352–367. 10.1107/S0907444912001308 PMC3322595 22505256

[bb2] Agirre, J., Atanasova, M., Bagdonas, H., Ballard, C. B., Baslé, A., Beilsten-Edmands, J., Borges, R. J., Brown, D. G., Burgos-Mármol, J. J., Berrisford, J. M., Bond, P. S., Caballero, I., Catapano, L., Chojnowski, G., Cook, A. G., Cowtan, K. D., Croll, T. I., Debreczeni, J. É., Devenish, N. E., Dodson, E. J., Drevon, T. R., Emsley, P., Evans, G., Evans, P. R., Fando, M., Foadi, J., Fuentes-Montero, L., Garman, E. F., Gerstel, M., Gildea, R. J., Hatti, K., Hekkelman, M. L., Heuser, P., Hoh, S. W., Hough, M. A., Jenkins, H. T., Jiménez, E., Joosten, R. P., Keegan, R. M., Keep, N., Krissinel, E. B., Kolenko, P., Kovalevskiy, O., Lamzin, V. S., Lawson, D. M., Lebedev, A. A., Leslie, A. G. W., Lohkamp, B., Long, F., Malý, M., McCoy, A. J., McNicholas, S. J., Medina, A., Millán, C., Murray, J. W., Murshudov, G. N., Nicholls, R. A., Noble, M. E. M., Oeffner, R., Pannu, N. S., Parkhurst, J. M., Pearce, N., Pereira, J., Perrakis, A., Powell, H. R., Read, R. J., Rigden, D. J., Rochira, W., Sammito, M., Sánchez Rodríguez, F., Sheldrick, G. M., Shelley, K. L., Simkovic, F., Simpkin, A. J., Skubak, P., Sobolev, E., Steiner, R. A., Stevenson, K., Tews, I., Thomas, J. M. H., Thorn, A., Valls, J. T., Uski, V., Usón, I., Vagin, A., Velankar, S., Vollmar, M., Walden, H., Waterman, D., Wilson, K. S., Winn, M. D., Winter, G., Wojdyr, M. & Yamashita, K. (2023). *Acta Cryst.* D**79**, 449–461.

[bb3] Al-Fakhrany, O. M. & Elekhnawy, E. (2023). *Arch. Microbiol.* **205**, 301. 10.1007/s00203-023-03639-0 PMC10406680 37550555

[bb5] Chen, V. B., Arendall, W. B., Headd, J. J., Keedy, D. A., Immormino, R. M., Kapral, G. J., Murray, L. W., Richardson, J. S. & Richardson, D. C. (2010). *Acta Cryst.* D**66**, 12–21. 10.1107/S0907444909042073 PMC2803126 20057044

[bb4] Collaborative Computational Project, Number 4 (1994). *Acta Cryst.* D**50**, 760–763.

[bb6] Cosconati, S., Forli, S., Perryman, A. L., Harris, R., Goodsell, D. S. & Olson, A. J. (2010). *Exp. Opin. Drug. Discov.* **5**, 597–607. 10.1517/17460441.2010.484460 PMC3083070 21532931

[bb7] Costa, L., das Graças Carvalho, M., La Guárdia Custódio Pereira, A. C., Teixeira Neto, R. G., Andrade Figueiredo, L. C. & Barros-Pinheiro, M. (2024). *Med. Princ. Pract.* **33**, 173–184. 10.1159/000538349 PMC11175606 38484713

[bb8] Eberhardt, J., Santos-Martins, D., Tillack, A. F. & Forli, S. (2021). *J. Chem. Inf. Model.* **61**, 3891–3898. 10.1021/acs.jcim.1c00203 PMC10683950 34278794

[bb9] Emsley, P. & Cowtan, K. (2004). *Acta Cryst.* D**60**, 2126–2132. 10.1107/S0907444904019158 15572765

[bb10] Emsley, P., Lohkamp, B., Scott, W. G. & Cowtan, K. (2010). *Acta Cryst.* D**66**, 486–501. 10.1107/S0907444910007493 PMC2852313 20383002

[bb11] Engh, R. A. & Huber, R. (2006). *International Tables for Crystallo­graphy*, Vol. F, 1st online ed., edited by M. G. Rossmann & E. Arnold, pp. 382–392. Chester: International Union of Crystallo­graphy.

[bb12] Gouet, P., Courcelle, E., Stuart, D. I. & Métoz, F. (1999). *Bioinformatics*, **15**, 305–308. 10.1093/bioinformatics/15.4.305 10320398

[bb13] Gouet, P., Robert, X. & Courcelle, E. (2003). *Nucleic Acids Res.* **31**, 3320–3323. 10.1093/nar/gkg556 PMC168963 12824317

[bb14] Greenwood, R. C. & Gentry, D. R. (2002). *J. Antibiot.* **55**, 423–426. 10.7164/antibiotics.55.423 12061551

[bb40] Kabsch, W. (2010). *Acta Cryst.* D**66**, 125–132. 10.1107/S0907444909047337 PMC2815665 20124692

[bb15] Kobayashi, T., Takimura, T., Sekine, R., Kelly, V. P., Kamata, K., Sakamoto, K., Nishimura, S. & Yokoyama, S. (2005). *J. Mol. Biol.* **346**, 105–117. 10.1016/j.jmb.2004.11.034 15663931

[bb16] Krissinel, E. & Henrick, K. (2004). *Acta Cryst.* D**60**, 2256–2268. 10.1107/S0907444904026460 15572779

[bb17] Lang, P. T., Brozell, S. R., Mukherjee, S., Pettersen, E. F., Meng, E. C., Thomas, V., Rizzo, R. C., Case, D. A., James, T. L. & Kuntz, I. D. (2009). *RNA*, **15**, 1219–1230. 10.1261/rna.1563609 PMC2685511 19369428

[bb18] Monobe, K., Taniguchi, H. & Aoki, S. (2024). *Curr. Comput. Aided Drug. Des.* **20**, 452–462. 10.2174/1573409919666230612120819 37309761

[bb19] Moustakas, D. T., Lang, P. T., Pegg, S., Pettersen, E., Kuntz, I. D., Brooijmans, N. & Rizzo, R. C. (2006). *J. Comput. Aided Mol. Des.* **20**, 601–619. 10.1007/s10822-006-9060-4 17149653

[bb20] Parkinson, G., Vojtechovsky, J., Clowney, L., Brünger, A. T. & Berman, H. M. (1996). *Acta Cryst.* D**52**, 57–64. 10.1107/S0907444995011115 15299726

[bb21] Qiu, X., Janson, C. A., Smith, W. W., Green, S. M., McDevitt, P., Johanson, K., Carter, P., Hibbs, M., Lewis, C., Chalker, A., Fosberry, A., Lalonde, J., Berge, J., Brown, P., Houge–Frydrych, C. S. V. & Jarvest, R. L. (2001). *Protein Sci.* **10**, 2008–2016. 10.1110/ps.18001 PMC2374228 11567092

[bb22] Rodríguez-Hernández, D., Vijayan, K., Zigweid, R., Fenwick, M. K., Sankaran, B., Roobsoong, W., Sattabongkot, J., Glennon, E. K. K., Myler, P. J., Sunnerhagen, P., Staker, B. L., Kaushansky, A. & Grøtli, M. (2023). *Nat. Commun.* **14**, 5408. 10.1038/s41467-023-41119-7 PMC10480161 37669940

[bb23] Rubio Gomez, M. A. & Ibba, M. (2020). *RNA*, **26**, 910–936. 10.1261/rna.071720.119 PMC7373986 32303649

[bb24] Sabbagh, P., Javanian, M., Koppolu, V., Vasigala, V. R. & Ebrahimpour, S. (2019). *Eur. J. Clin. Microbiol. Infect. Dis.* **38**, 1035–1045. 10.1007/s10096-019-03502-5 30734129

[bb25] Serbzhinskiy, D. A., Clifton, M. C., Sankaran, B., Staker, B. L., Edwards, T. E. & Myler, P. J. (2015). *Acta Cryst.* F**71**, 594–599. 10.1107/S2053230X15004677 PMC4427170 25945714

[bb26] Simpkin, A. J., Simkovic, F., Thomas, J. M. H., Savko, M., Lebedev, A., Uski, V., Ballard, C., Wojdyr, M., Wu, R., Sanishvili, R., Xu, Y., Lisa, M.-N., Buschiazzo, A., Shepard, W., Rigden, D. J. & Keegan, R. M. (2018). *Acta Cryst.* D**74**, 595–605. 10.1107/S2059798318005752 PMC6038384 29968670

[bb27] Stacy, R., Begley, D. W., Phan, I., Staker, B. L., Van Voorhis, W. C., Varani, G., Buchko, G. W., Stewart, L. J. & Myler, P. J. (2011). *Acta Cryst.* F**67**, 979–984. 10.1107/S1744309111029204 PMC3169389 21904037

[bb28] Stefanska, A. L., Coates, N. J., Mensah, L. M., Pope, A. J., Ready, S. J. & Warr, S. R. (2000). *J. Antibiot.* **53**, 345–350. 10.7164/antibiotics.53.345 10866215

[bb29] Studier, F. W. (2005). *Protein Expr. Purif.* **41**, 207–234. 10.1016/j.pep.2005.01.016 15915565

[bb30] Sun, J., Lv, P. C. & Zhu, H. L. (2017). *Expert Opin. Ther. Pat.* **27**, 557–564. 10.1080/13543776.2017.1273350 27977303

[bb31] Trott, O. & Olson, A. J. (2010). *J. Comput. Chem.* **31**, 455–461. 10.1002/jcc.21334 PMC3041641 19499576

[bb32] Wang, X. D., Wei, W., Wang, P. F., Yi, L. C., Shi, W. K., Xie, Y. X., Wu, L. Z., Tang, N., Zhu, L. S., Peng, J., Liu, C., Li, X. H., Tang, S., Xiao, Z. P. & Zhu, H. L. (2015). *Bioorg. Med. Chem.* **23**, 4860–4865. 10.1016/j.bmc.2015.05.026 26048027

[bb33] Williams, C. J., Headd, J. J., Moriarty, N. W., Prisant, M. G., Videau, L. L., Deis, L. N., Verma, V., Keedy, D. A., Hintze, B. J., Chen, V. B., Jain, S., Lewis, S. M., Arendall, W. B., Snoeyink, J., Adams, P. D., Lovell, S. C., Richardson, J. S. & Richardson, J. S. (2018). *Protein Sci.* **27**, 293–315. 10.1002/pro.3330 PMC5734394 29067766

[bb41] Winn, M. D., Ballard, C. C., Cowtan, K. D., Dodson, E. J., Emsley, P., Evans, P. R., Keegan, R. M., Krissinel, E. B., Leslie, A. G. W., McCoy, A., McNicholas, S. J., Murshudov, G. N., Pannu, N. S., Potterton, E. A., Powell, H. R., Read, R. J., Vagin, A. & Wilson, K. S. (2011). *Acta Cryst.* D**67**, 235–242. 10.1107/S0907444910045749 PMC3069738 21460441

[bb34] Xin, Y., Li, W. & First, E. A. (2000). *J. Mol. Biol.* **303**, 299–310. 10.1006/jmbi.2000.4126 11023794

[bb35] Yaremchuk, A., Kriklivyi, I., Tukalo, M. & Cusack, S. (2002). *EMBO J.* **21**, 3829–3840. 10.1093/emboj/cdf373 PMC126118 12110594

[bb36] Zhu, N., Lin, Y., Li, D., Gao, N., Liu, C., You, X., Jiang, J., Jiang, W. & Si, S. (2015). *J. Antimicrob. Chemother.* **70**, 2287–2294. 10.1093/jac/dkv110 25957580

