# Supplementary material for: Crystal structure of tyrosine-tRNA synthetase from Helicobacter pylori G27
Source: Acta Crystallogr F Struct Biol Commun. 2026 Oct 23;82(Pt 11):369–75. doi: 10.1107/S2053230X26010253 (PMC13717830; doi:10.1107/S2053230X26010253)
Supplement: Supplementary file 1 [file f-82-00369-sup1.pdf]

STRUCTURAL BIOLOGY COMMUNICATIONS

Volume 82 (2026)

Supporting information for article:

Crystal structure of tyrosine-tRNA synthetase from *Helicobacter pylori* G27

David Igbalajobi, Jason E. Taylor, Raymond Gao, Jesuferanmi Ayanlade, Rachael Zigweid, Sandhya Subramanian, Hannah Udell, Thomas E. Edwards, Jan Abendroth, Donald Lorimer, Peter J. Myler, Bart Staker, Edmund Moses Ndip and Oluwatoyin A. Asojo

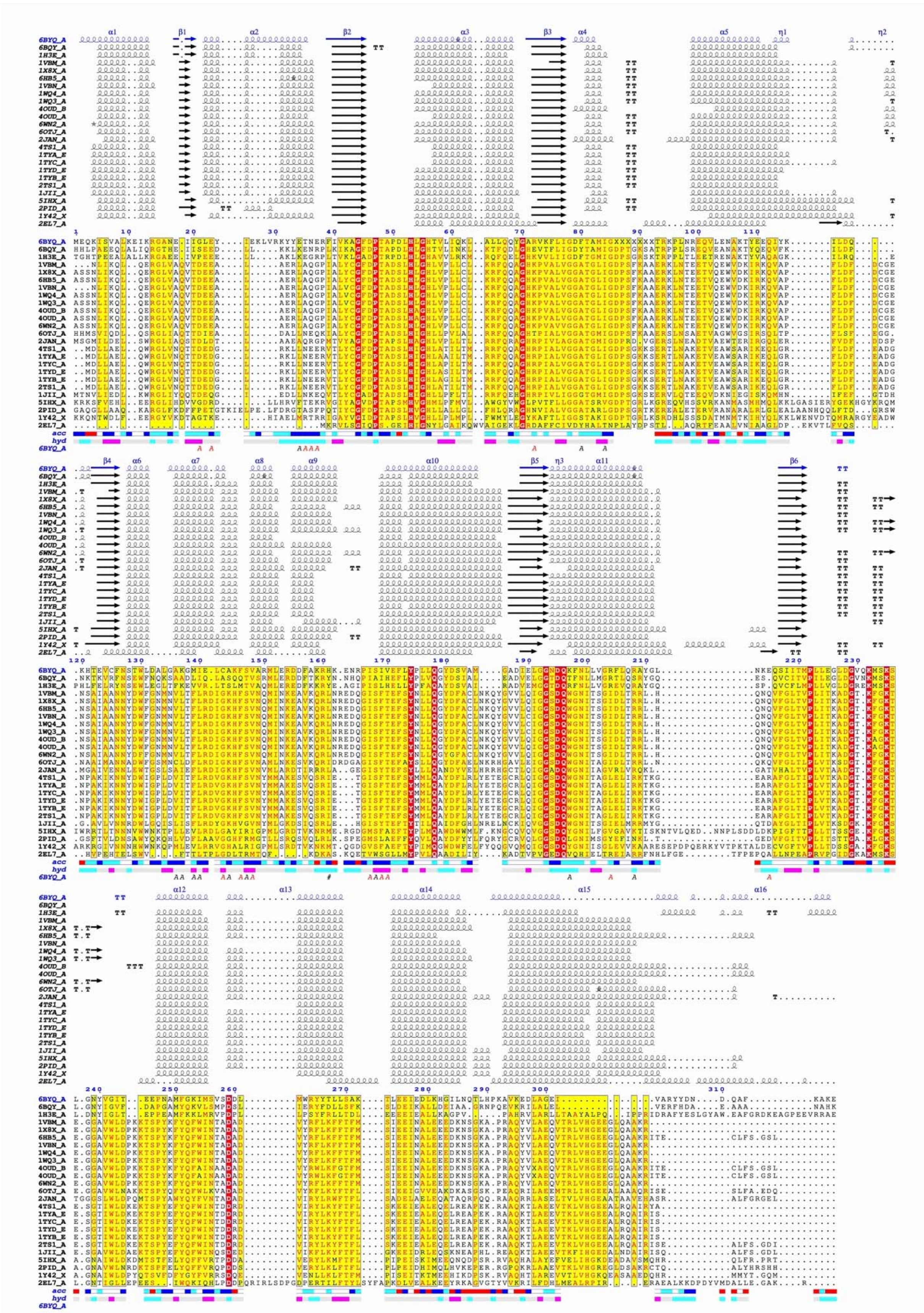

Figure S1 ENDScript analysis reveals the nearest structural neighbors of *Hp*TyrRS. Identical and conserved residues are highlighted in red and yellow, respectively. The different secondary structure elements shown are alpha helices (α), 3_{10}-helices (η), beta strands (β), and beta turns (TT) (Gouet *et al.*, 2003, Gouet *et al.*, 1999).
